# Supplementary material for: A New SMAD4 Splice Site Variant in a Three-Generation Italian Family with Juvenile Polyposis Syndrome
Source: Diagnostics (Basel). 2022 Nov 4;12(11):2684. doi: 10.3390/diagnostics12112684 (PMC9689379; doi:10.3390/diagnostics12112684)
Supplement: Supplementary file 1 [file diagnostics-12-02684-s001.zip › diagnostics-1974141-supplementary.pdf]

Gene	Reference sequence	Chr	Locus		Gene uniformity
			start	end	
ATM	NM_000051.3	chr11	108117686	108236240	97.43%
PALB2	NM_024675.3	chr16	23614775	23649278	100%
MRE11A	NM_005591.3	chr11	94203632	94163157	100%
RAD50	NM_005732.3	chr5	131977865	131944422	100%
BARD1	NM_000465.3	chr2	215593395	215646238	100%
NBN	NM_002485.4	chr8	90990443	90982790	99.22%
BRIP1	NM_032043.2	chr17	59763192	59858371	96.28%
RAD51C	NM_058216.2	chr17	56809840	56787356	100%
RAD51D	NM_001142571.2	chr17	33434380	33446637	99.99%
STK11	NM_000455.4	chr19	1220575	1223176	94.69%
MSH2	NM_000251.2	chr2	47630326	47637516	99.99%
MLH1	NM_000249.3	chr3	37053497	37070428	100%
MSH6	NM_000179.2	chr2	48010368	48033795	99.49%
PMS2	NM_000535.6	chr7	6045518	6037059	100%
EPCAM	NM_002354.2	chr2	47596640	47606198	95.35%
MUTYH	NM_001128425.1	chr1	45798430	45800188	93.4%
RECQL1	NM_032941.2	chr12	21627770	21652509	91.39%
TP53	NM_000546.5	chr17	7576532	7578294	99.35%
PTEN	NM_000314.6	chr10	89711870	89725234	82.32%
CHEK2	NM_007194.3	chr22	29091693	29121117	98.88%
CDH1	NM_004360.4	chr16	68862072	68856133	98.34%
CDK4	NM_000075.3	chr12	58143232	58145130	100%
CDKN2A	NM_001195132.1	chr9	21970896	21968775	99.56%
SMAD4	NM_005359.5	chr18	48591788	48593562	100%
APC	NM_000038.5	chr5	112111321	112163708	100%

Supplementary Table S1: Custom Panel were designed with Ampliseq Designer V.7.0 (https://ampliseq.com/protected/startPage.action); features: 25 genes, 610 amplicons with size range 125-275 bp and size: 113.732 Kb; Reference Sequence from https://www.ncbi.nlm.nih.gov/refseq/; chr: chromosome
